# Burden of disease and cost of illness of infants less than 6 months of age hospitalised with respiratory syncytial virus in Denmark – a 10-year national register-based study

**DOI:** 10.1186/s12879-024-09975-w

**Published:** 2024-10-03

**Authors:** Marie-Louise von Linstow, Jan Håkon Rudolfsen, Jens Olsen, Mette Skovdal, Nina Breinholt Staerke

**Affiliations:** 1 Department of Pediatrics and Adolescent Medicine, Copenhagen University Hospital, Rigshospitalet, Blegdamsvej 9, Copenhagen, DK-2100 Denmark; 2 EY, Frederiksberg, Denmark; 3 Pfizer Denmark ApS, Ballerup, Denmark; 4 Department of Infectious Diseases, Aarhus University Hospital, Aarhus, Denmark

**Keywords:** Respiratory syncytial virus, Real world data, Healthcare utilisation, Cost

## Abstract

**Background:**

Respiratory syncytial virus (RSV) is the most common cause of hospitalisation in infants aged ≤ 6 months in Western countries. Nearly 1,500 infants under six months of age are hospitalised with RSV annually in Denmark. This nationwide study describes the healthcare resource utilisation and costs related to RSV hospitalisation in this vulnerable age group.

**Methods:**

RSV cases were identified in the Danish National Patient Register. Infants were included if they at the age of 0–5 months had a (1) respiratory related hospital admission (duration > 12 h), (2) within 10 days of a positive RSV test, (3) between January 2013 and December 2022. Each case was matched with five individuals never diagnosed with RSV on age, sex, region of residence, birth (pre/full term), number of siblings < 7 years old, and parents’ education. An episode of RSV was defined as the seven days prior to hospitalisation to 30 days after initial hospitalisation. Study outcomes included contacts with hospital and primary care, and total healthcare costs defined as the sum cost of hospital care, primary care, and prescription medicine. Cost and contacts attributable to RSV was calculated in a diff-in-diff framework, as the difference between case and reference group.

**Results:**

The study population comprised of 8,428 RSV cases and 41,725 reference individuals. Cases generated 1.58 (*p* < 0.001) attributable inpatient contacts, 0.84 (*p* < 0.001) outpatient contacts, and 1.19 (*p* < 0.001) primary care contacts during their RSV episode. An additional 0.6 (*p* < 0.001) inpatient, 1.08 (*p* < 0.001) outpatient and 2.42 (*p* < 0.001) primary care contacts were attributed to RSV in the year following the RSV episode. Total cost of an RSV episode was EUR 2,997 (*p* < 0.001) with an additional EUR 1,428 (*p* < 0.001) in the following year.

**Conclusion:**

RSV hospitalisations of infants are associated with substantial healthcare utilisation and costs. The same pattern was observed in the year following the RSV episode. If the new RSV prevention options are introduced nationwide, the overall burden of RSV is expected to be substantially reduced in the future.

## Introduction

Respiratory syncytial virus (RSV) is the most common cause of hospitalisation in infants aged ≤ 6 months in Western countries. The clinical presentation of RSV disease ranges from mild upper respiratory tract symptoms, to potentially life-threatening lower respiratory infection [1]. It is estimated that RSV is the cause of 30 million acute respiratory infections in children each year, resulting in more than 60,000 deaths worldwide [2, 3].

RSV is a major public health concern with significant treatment costs. Due to the seasonality of infections, it causes an uneven pressure on the healthcare system, making it difficult to optimize resource allocation to the healthcare sector. At the age of two, the majority of children in Denmark will have had an RSV infection, with approximately one-third of infants with RSV developing lower respiratory tract symptoms [4]. Between 2010 and 2016, 1.6% of infants in Denmark were hospitalised due to RSV within the first six months of life. For comparison, 0.1% were hospitalized due to influenza [3, 5]. Globally, RSV accounts for around 3.6 million lower respiratory tract hospitalisations each year in young children, with 1.4 million of these occurring in children aged ≤ 6 months [6]. In Western countries, the duration of hospitalisations due to RSV range from 2 to 11 days and 2–12% of cases require intensive care treatment [7].

Moreover, in the US, RSV is associated with 15% of all paediatric office visits for respiratory infections between November and April [8]. Similarly, it is estimated that in 2016–2022, 16.3% of 0-5-year-old children hospitalised with respiratory disease in Denmark had RSV. Of the patients admitted with RSV about 59% were less than 6 months old [9].

A systematic review of the published literature and assembled unpublished data identified the overall costs related to management of acute lower respiratory tract infection episodes caused by RSV from 1987 to 2017 in young children [10]. The review reported an average cost per RSV episode of €3,452 (95% CI: 3,265-3,639) and €299 (95% CI: 295–303) for inpatient and outpatient management without follow-up, respectively. The costs increased to €8,591 (95% CI: 8,489-8,692) and €2,191 (95% CI: 2,190-2,192) when including 2 years of follow-up after the initial RSV event [10]. A similar study from Denmark found that the annual hospital cost related to RSV cases requiring inpatient hospitalization was EUR 4.1 million [11] while an Icelandic study reported annual direct and indirect cost of RSV in children younger than 5 years of EUR 4,3 million [12].

While the aforementioned review and the study by Jepsen et al. [11] have estimated the hospital cost of RSV in infants, they did not include cost of drugs and primary care, nor was a RSV-free control population included to estimate the attributable cost of RSV. As infants will generate healthcare costs, even without RSV, it is important to identify what proportion of the generated cost is attributable to RSV. This is particularly important now, as several new opportunities for prevention through long-lasting antibodies and maternal vaccination have demonstrated positive effects in clinical trials [13, 14].

Therefore, this study assesses the burden of RSV in infants younger than six months by estimating the attributable cost of RSV in Denmark between 2013 and 2022. The study identifies infants hospitalised with RSV in Danish national registers and constructs a matched reference population. The difference in cost and healthcare resource utilisation between the RSV and reference group is then defined as the cost attributable to RSV. The results will be key in providing stakeholders with updated information when evaluating RSV preventative alternatives.

## Materials and methods

This study is a retrospective register study applying Danish national health and administrative registers for the period 1 January 2013 to 31 December 2022. The registers include The Civil Registration System (CRS) for information on age, sex and residence region [15]; The Danish Microbiology Database (MiBa); the National Patient Register (NPR) for information on ICD-10 codes and procedure codes, including codes for specific treatments, and information on Diagnosis Related Groups (DRG) tariffs [16, 17]; the Register of Selected Chronic Diseases (RUKS) for information on asthma; the National Health Insurance Service Register for information on contacts, services received and fees in the primary healthcare sector [18]; the Cause of Death Register for information on date of death [19]; the Register of Medicinal Product Statistics for information on expedition date and prices for all prescription drugs sold in Danish community pharmacies [20]; The Danish Education Register for information on parents education [21].

Each register contains individual level observations. Individuals can be identified through a unique 10-digit personal identification number, making it possible to securely merge data from the different registers. Furthermore, individuals are listed with mothers’ and fathers’ personal identification number in the civil registration system, allowing for merging of intergenerational data.

### Study population

 RSV cases were identified using NPR. Individuals with RSV-related hospital admissions were cross-referenced with MiBa, a national microbiology register used for the surveillance of infectious diseases. If a positive RSV test in MiBa was observed within 10 days (+/-) of a hospital admission where an RSV related diagnoses code was listed as primary or secondary diagnosis, the individual was classified as an RSV case. The 10 days leeway between hospital admission and positive RSV test was to allow for potential frictions between date registrations in the two different registers. The criteria for RSV related diagnoses are presented in Table 1. As most of the infants with RSV in Denmark are admitted in their first six months [5] and the majority of infants are admitted before the age of one year, only RSV cases who were younger than six months at time of hospital admission were included in the base case scenario. In a sensitivity analysis, this inclusion criterion was expanded to including all cases who were younger than 12 months at time of hospital admission.

**Table 1:** Respiratory hospital admissions defined by ICD-10 codes

ICD-10 code/chapters	Description
B97	Viral agents as the cause of disease classified to other chapters
J00 – J06	Acute upper respiratory infections
J09 – J18	Influenza and pneumonia
J20 – J22	Other acute lower respiratory infections
J30 – J39	Other diseases of the upper respiratory tract
R50	Fever of other and unknown origin
R56	Convulsions, not elsewhere classified

Each RSV case was subsequently pairwise matched with five individuals who were not observed with an RSV hospitalisation to constitute a reference group. The five control individuals were unique for the RSV case, but the controls could be matched with multiple RSV cases – i.e., matching with replacement. The matching covariates were age (+/- 30 days), sex, region of residence, birth (pre/full term), number of siblings < 7 years old, and parents’ education (highest education of any parent). Each individual in the reference group was assigned a pseudo index date to reflect the age at index of the matched RSV case.

### Outcomes

The case and reference population were compared in terms of use and costs of primary and secondary healthcare services as well as use of prescription drugs. The use and cost of healthcare services attributable to RSV was calculated as the mean difference between the case and reference group.

Hospital contacts were described as inpatient and outpatient contacts, based on length of stay. Hospital contacts < 12 h were classified as outpatient contacts. Hospital contacts ≥ 12 h were classified as inpatient contacts. Cost of hospital care were calculated based on DRG tariffs. The DRG tariffs are a function of the patients age, diagnosis, length of stay, procedures and treatments provided in a hospital contact. Based on economic and activity data from all Danish hospitals, the tariffs are estimated each year to reflect updated national mean costs for inpatient and outpatient hospital contacts.

Cost of primary care were estimated based on the reimbursement fees to primary care practitioners (general practitioners (GPs) and privately practicing specialists). Cost of prescription medications were based on the pharmacy sales prices. All costs associated with prescription medications and primary care contacts financed by the government, including routine child examinations, were included in the analysis.

All prices were converted to 2023 prices through the consumer price index provided by Statistics Denmark. Danish currency (DKK) was transformed to EUR using the conversion rate 1 EUR = 7.5 DKK.

Finally, previous studies have demonstrated an association between RSV hospitalisation in infancy and the development of asthma – with the causal link being unaccounted for. To place the results of the study in context, we provide an overview of asthma in the RSV case and control population, and for the parents of RSV cases and parents of the controls. Asthma was defined in the register of select chronic diseases, where an algorithm depending on age, hospital contacts and collection of prescription medication is used to determine asthma [22]. Any registration of asthma between hospital contact until end of follow-up was identified in the RSV and control population, while any registration of asthma since 1994 onwards in the adult population was used to determine prevalence of asthma.

### Ethical statement

The study was conducted in accordance with legal and regulatory requirements and with scientific purpose, value, and rigour. The study followed the generally accepted research practices described in International Ethical Guidelines for Epidemiological Studies, issued by the Council for International Organizations of Medical Sciences (CIOMS).

Data was stored on Statistics Denmark’s research computers – an environment maintained by Statistics Denmark, fulfilling all requirements for storage and handling of personal sensitive data. Data was accessed through a log-in solution using a two-step validation and administrative access control. While data was available at individual level, all personal identification numbers were encrypted by Statistics Denmark before data was made available to the researchers.

No data were sent from the server to open environments. Only aggregated tables containing at least five observations were exported as part of the research process.

### Statistical analysis

An RSV episode was defined as the seven days prior to hospitalisation to 30 days after initial hospitalisation. Furthermore, we studied the year following the RSV episode, divided into two six-month periods.

The data was analysed in a difference-in-difference with variation in treatment timing framework [23], using a linear ordinary least square regression model. The difference-in-difference approach is frequently used in instances where it is unethical or not possible to conduct a randomized control trial. The method can identify either the within-group differences (before and after event – in this case RSV event), in outcome, or between-group differences (difference between case and control group). In this study, the focus was to identify the between-group differences – i.e., differences attributable to the RSV event. When identifying the between-group differences, the underlying assumption is that the two groups are equal prior to the event (parallel trend assumption).

The young age of the study participants in this instance makes it difficult to assess the similarity between the RSV cases and their controls prior to the RSV event. The parallel trend assumption is therefore, in theory, applied by matching on both health determinants at birth, and socioeconomic position of the parents. Therefore, if no event occurs, the two groups will remain equal over time. Any differences observed between the groups will therefore be attributable to the event of interest. To ensure a balanced analysis in all periods, each reference individual was weighted. The weights were calculated such that the sum of weights for controls to each RSV case was equal to 1.

Statistically significant differences between the two groups were evaluated by testing the difference in the mean for each group in each period. A two-sided t-test was applied with a significance level of 0.05. When analysing the differences in asthma between RSV cases and controls, or the parents of the two groups, a chi-square test was conducted.

As a sensitivity test, the inclusion criteria were expanded to all individuals younger than one year at time of RSV hospitalisation, in the same analytical framework. An analysis identifying RSV cases only using ICD-10 codes was also conducted. Furthermore, the outcomes were evaluated for up to nine years after the initial RSV episode.

## Results

**Fig. 1:**
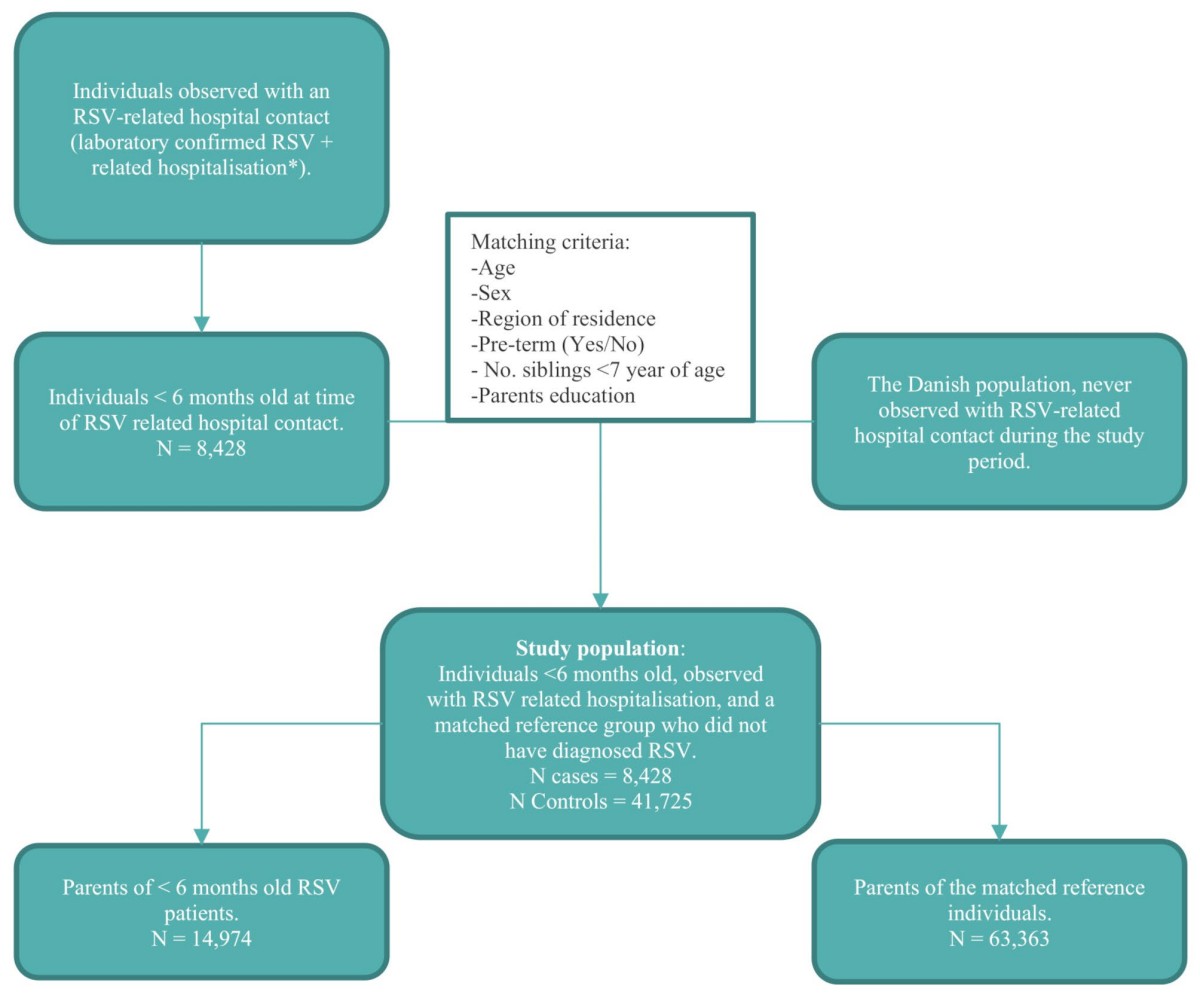
Flowchart visualizing the identification of the two study populations. Patients with RSV, the controls and the parents *RSV related hospitalisation defined as a hospital admission with any of the following ICD-10 codes: B97, J00 – J06, J09 – J18, J20 – J22, J30 – J39, R50, R56.0

A total of 8,428 infants were identified with an RSV hospitalisation during the study period, matched with 41,725 controls. A flowchart for identifying cases and controls is presented in Fig. 1, while baseline characteristics are presented in Table 2.

**Table 2:** Summary statistics at baseline

	Cases	Controls
*N*	8,428	41,725
Age (infants, days), mean (SD)	73 (45)	73 (45)
Age (parents, years), mean (SD)	33 (5)	33 (5)
Annual income, parents (euro, 2021-prices), mean (SD)	60,765 (281,503)	59,058 (79,539)
Sex		
Female, n (%)	3,746 (44%)	18,545 (44%)
Male, n (%)	4,682 (56%)	23,180 (56%)
Region		
The Capital Region of Denmark, n (%)	3,660 (43%)	18,125 (43%)
Central Denmark Region, n (%)	1,181 (14%)	5,871 (14%)
The North Denmark Region, n (%)	1,011 (12%)	4,967 (12%)
Region Zealand, n (%)	879 (10%)	4,334 (10%)
Region of Southern Denmark, n (%)	1,697 (20%)	8,428 (20%)
Education (parents)		
Bachelor or equivalent, n (%)	3,879 (23%)	21,438 (26%)
Elementary school, n (%)	3,291 (20%)	12,270 (15%)
Not in the education registers, n (%)	808 (5%)	3,722 (4%)
Master or higher, n (%)	3,304 (20%)	15,441 (19%)
Secondary education, n (%)	4,688 (28%)	25,993 (31%)
Short cycle tertiary, n (%)	885 (5%)	4,586 (5%)
Parents with asthma	2,111 (13.4%)	8,100 (10.4%)

The cases were on average 73 days old at time of hospitalisation, with parents in their early thirties. More males than females were admitted to the hospital with RSV. After matching, the socioeconomic background did not differ with regards to income. We did, however, observe a significantly higher incidence of asthma in parents of the RSV cases (13.4%), compared with parents in the reference population (10.4%) (*p* < 0.001).

### Healthcare resource use and comorbidities

**Fig. 2:**
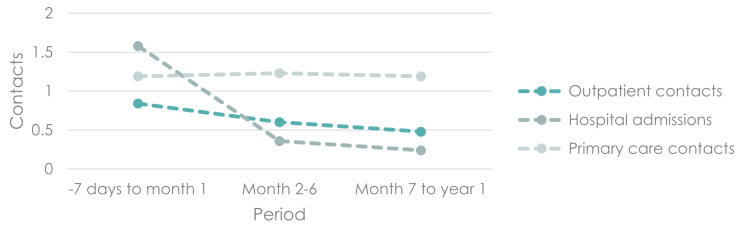
Hospital and primary care contacts attributable to RSV Note: Attributable contacts calculated as mean number of contacts in RSV population minus mean number of contacts in reference group

Healthcare resource use attributable to RSV is presented by group in Fig. 2 with nominal contacts for RSV cases, controls and attributable cost of RSV values presented in Table 3. RSV cases had 1.65 inpatient contacts during their RSV episode. These hospitalisations include the hospital contact required in the inclusion criteria. The individuals in the reference population had on average 0.07 inpatient contacts during the same period. Hence, 1.58 inpatient contacts can be attributed to the RSV episode. In the year following the RSV episode, an additional 0.6 inpatient hospital contacts could be attributed to RSV.

**Table 3:** Mean nominal contacts for RSV cases and controls, and contacts attributable to RSV

		-7 days to month 1	Month 2–6	Month 7 to year 1
Outpatient contacts	RSV	1.09 (1.04–1.13)	1.03 (0.98–1.08)	0.93 (0.88–0.98)
	Control	0.25 (0.2–0.29)	0.43 (0.38–0.47)	0.45 (0.4–0.5)
	Attributable	0.84 (0.77–0.9)	0.6 (0.54–0.67)	0.48 (0.41–0.55)
Inpatient contacts	RSV	1.65 (1.63–1.66)	0.46 (0.45–0.48)	0.4 (0.39–0.41)
	Control	0.06 (0.05–0.08)	0.11 (0.09–0.12)	0.16 (0.15–0.18)
	Attributable	1.58 (1.57–1.6)	0.36 (0.34–0.38)	0.24 (0.22–0.26)
Primary care contacts	RSV	2.39 (2.31–2.46)	4.76 (4.68–4.84)	5.4 (5.32–5.48)
	Control	1.19 (1.12–1.27)	3.53 (3.45–3.61)	4.21 (4.14–4.29)
	Attributable	1.19 (1.09–1.29)	1.23 (1.12–1.34)	1.19 (1.08–1.3)

Note: Attributable contacts calculated as mean difference between RSV cases and their reference individuals

Similarly, RSV cases had on average 1.09 outpatient contacts during the RSV episode, compared to 0.25 contacts among the reference group – i.e., 0.84 outpatient contacts were identified as attributable to RSV. In the year following the RSV episode, 1.08 attributable outpatient contacts were identified.

Finally, RSV cases had 2.39 contacts with their primary care physician (GP or on-call GP) during the RSV episode, compared to 1.2 contacts in the reference population, resulting in 1.19 primary care contacts being attributed to RSV. In the year following the RSV episode, we observed 2.42 contacts attributable to RSV.

Considering the time from hospitalisation to end of follow-up (31 December 2022), we observed 7.1% of RSV cases being diagnosed with bronchitis, versus 2.3% of the reference population (*p* < 0.001). Furthermore 0.89% of the RSV cases and 0.57% of reference individuals were later diagnosed with asthma (*p* = 0.001). Both comorbidities were identified more than 30 days after the RSV episode.

### Cost of healthcare services

**Fig. 3:**
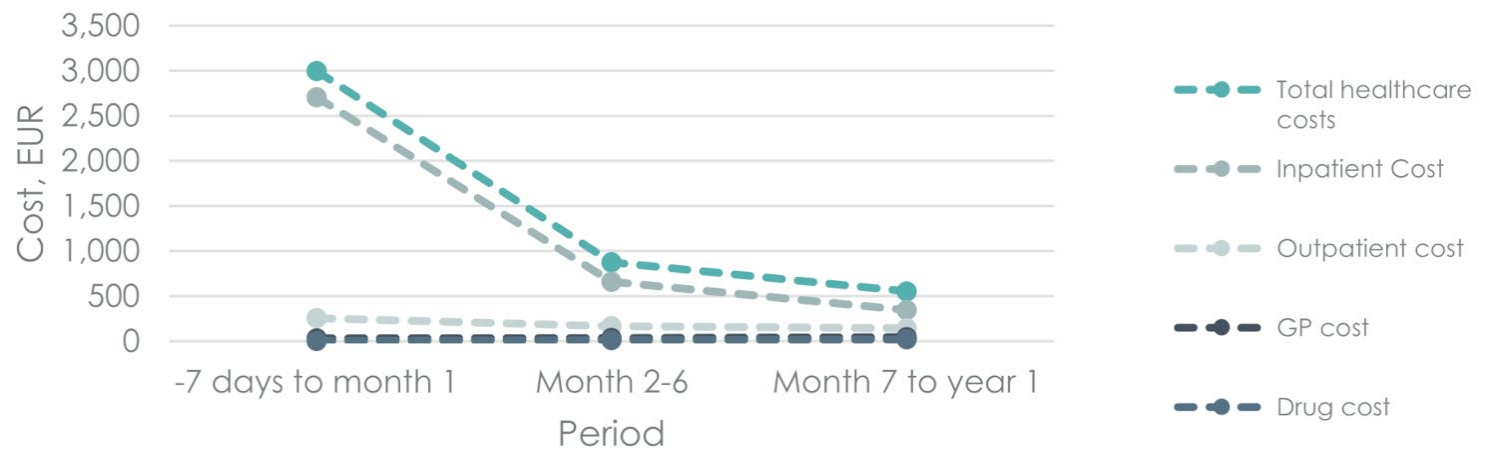
Healthcare costs attributable to RSV Note: Attributable cost calculated as mean cost of RSV minus mean cost in reference group

Attributable cost of RSV by cost category is presented in Fig. 3, with nominal cost for RSV cases, controls and attributable cost of RSV values presented in Table 4. Total cost of an RSV episode was EUR 3,201, with EUR 2,997 being estimated to the attributable cost of RSV. In the following year after the RSV episode, an additional EUR 1,428 was generated as attributable cost.

**Table 4:** Mean nominal cost of RSV and controls, and attributable cost to RSV by cost category

		-7 days to month 1	Month 2–6	Month 7 to year 1
Total Cost	RSV	3,201 (3,041 − 3,361)	1,246 (1,074 − 1,418)	976 (804-1,148)
	Control	204 (44–364)	372 (200–543)	423 (251–595)
	Attributable to RSV	2,997 (2,770-3,223)	875 (632-1,117)	553 (309–796)
Inpatient Cost	RSV	2,821 (2,762-2,881)	808 (745–872)	490 (426–553)
	Control	114 (55–173)	147 (84–211)	145 (81–209)
	Attributable to RSV	2,707 (2,624-2,791)	661 (571–751)	345 (255–435)
Outpatient cost	RSV	308 (172–444)	268 (122–414)	262 (116–408)
	Control	54 (-82-190)	103 (-43-249)	118 (-28-265)
	Attributable to RSV	254 (62–446)	164 (-42-371)	144 (-63-351)
GP cost	RSV	65 (61–68)	146 (142–150)	184 (180–188)
	Control	33 (29–37)	110 (106–114)	138 (134–142)
	Attributable to RSV	31 (26–37)	37 (31–42)	46 (40–51)
Drug cost	RSV	7 (2–12)	24 (19–29)	40 (35–45)
	Control	3 (-2-8)	11 (6–16)	21 (16–26)
	Attributable to RSV	4 (-3-10)	13 (6–20)	19 (12–26)

Note: Attributable cost calculated as mean difference between RSV cases and their reference individuals

Inpatient hospitalization was the biggest source of the cost of RSV, making up EUR 2,821 of the EUR 3,201 corresponding to 88% of the total cost. The attributable cost of RSV related to inpatient hospitalization was EUR 2,707, with an additional EUR 1,006 attributable cost in the year following the RSV event.

Outpatient hospital cost generated EUR 254 in attributable cost while GP attributable cost was found to be EUR 31. Drug cost only accounted for 4 EUR in attributable cost during the RSV event (*p* > 0.05), and EUR 32 in the year following the RSV event.

### Sensitivity analysis

The results were consistent when expanding the inclusion criteria to include all hospitalised RSV cases under one year of age, where the total attributable cost was estimated to be EUR 2,926. When including all infants with an RSV specific ICD-10 code, the cost estimate in the RSV episode increased slightly. However, the costs in the year following the RSV decreased. Hence, the overall estimate did not influence the inference from the study. In the analysis of long-term outcomes, significant differences in total costs were found in the two years after the RSV episode.

## Discussion

This study identified the attributable cost of RSV hospitalisation in infants less than six months of age over a 10-year period in Denmark. Cases were identified through a national database for microbiology testing from 2013 to 2022 in combination with an RSV related hospital contact. A control population was identified in the general Danish population and matched on key covariates. An RSV event was defined as 7 days before to 30 days after the initial hospitalisation. The attributable cost of an RSV event in infants aged ≤ 6 months was EUR 2,997. Inpatient hospital care was the largest cost category, constituting 90% of the attributable costs. The results were robust to age inclusion criteria of the participants, as in infants aged ≤ 12 months, the total excess cost was EUR 2,926. Furthermore, the inference did not change when identifying RSV cases based on selected ICD-10 codes. The attributable cost of RSV was significantly higher for all included categories of healthcare, as well as additional inpatient and outpatient hospital contacts. We are not aware of previous studies including a control group to be able to estimate the total cost attributable to the RSV disease.

Statens Serum Institut, which is a national institute under the auspices of the Danish Ministry of Health, reported 1,447 RSV hospitalisations in Denmark for infants 0–5 months of age in the 2022/2023 season. Extrapolating from these numbers the attributable cost of RSV episodes in infants 0–5 months of age in Denmark was EUR 4.3 million in the 2022/2023 season. Additionally, higher cost of care in the year following the RSV episode was observed, generating an extra EUR 2.1 million in attributable cost. This corresponds well with the estimate from Jepsen et al. 2018 [11] of hospitalisation-associated costs of RSV in Denmark to be EUR 4.1 million pr year. Note that these are attributable costs of RSV, where the costs associated with unrelated healthcare services observed in the control population is subtracted.

For context, the cost of RSV hospitalisations in the Netherlands the was EUR 3.8 million in 2021/2022 season [24]. Moreover, the cost associated with RSV, if including non-hospitalized cases is likely larger. In Norway, which has comparative healthcare system and population size to Denmark, it was found that based on 13 517 RSV cases, of which 1572 were hospitalizations, the cost was EUR 8 million annually [25] in children under five years old. Lastly, in Germany, it is estimated that the mean cost of an RSV episode requiring hospitalisation was EUR 3,286 [26] compared to the EUR 3,201 found in this study.

We found that parents of RSV cases had higher incidence of asthma than parents of the reference group (13.4% vs. 10.4%), and in later childhood a significantly higher incidence of asthma was observed in the RSV population compared to the reference group (0.89% vs. 0.57%). While the incidence was small, it should be seen in context of the difficulty of diagnosing asthma in young children. Asthma is considered a hereditary illness through genetics or socioeconomics [27]. The association between RSV hospitalisation in infancy and asthma is well documented [28], but the causal relationship is unclear [29]. Our findings suggest, however, that presence of respiratory illnesses in parents could be considered when identifying infants with higher risk of severe RSV infections in infancy.

Thus, RSV poses a substantial burden on infants and children, their families and society as a whole and the seasonality constitutes an uneven and especially high burden in the winter half-year. However, different preventive options are now available implying that the burden of RSV can be reduced markedly in the future [13, 14].

A strength of this study was the use of pair-wise matching where specific reference individuals for each case could be identified. The reference individuals were compared pairwise with the specific RSV case from the same date the case was hospitalised allowing a comparison of the outcomes following the RSV hospitalisation.

A limitation of the analysis presented here was the extensive use of national registers which means that the study relies on the accuracy and completeness of the registers. However, the quality of records in the national Danish registers is generally assessed to be of high quality. In addition, due to the reliance on retrospective data, no conclusions on causal relations can be made from the analysis presented. However, this was in line with the descriptive and explorative purpose of this study.

The NPR was reformed during the study period (from 2018 to 2019). This resulted in a change in the format of the register. While this change was accounted for in the analysis, we cannot rule it out as a source of bias in the study. Particularly, we allowed for up to 30 days difference in date of birth between cases and their respective controls. This difference in birth date may result in cases and controls being observed in different years (December and January in the following year). While this might influence the results for all years included in the study, it is particularly sensitive in association with the reform of the registers. However, the focus of the study is the between-group difference. Since the changes in the registers influence both the RSV and the case population, the implications will be limited.

RSV cases not resulting in a hospital contact cannot be identified systematically and might appear in the reference population. However, as the study objective was to estimate the burden associated with RSV inpatient hospitalisations and compare this to the general background population this was assessed to constitute a minor inaccuracy in this study. In addition, some of the mild cases of respiratory tract infections are handled in the primary sector by the GP where RSV tests are rarely performed. Consequently, there is the potential for some individuals in the reference group to have milder course of RSV, not requiring hospitalisation. Despite this potential contaminated reference population, the study results represent the healthcare cost treating RSV. In most cases, parents will not be able to distinguish RSV from a common cold. Therefore, cases not recorded at the hospital will result in limited healthcare costs.

The difference-in-difference method applied in the study assumes parallel trend prior to event. With the young age of the study participants, we could not reliably assure this assumption in the analysis. However, through the matching process, we have included factors which should theoretically assure parallel trends between the two groups. Household structure and parents’ education were included to ensure similar socioeconomic status of cases and controls, while sex and birth preterm/full term were included as they are strong predictors of health at birth. Granted, it would be beneficial to have a more granular definition of preterm/full term birth. However, when more granular definitions were implemented, it resulted in exclusion of cases. Despite matching on these influential covariates, we cannot rule out the possibility of remaining unobserved residual confounding.

Diagnostic procedures for identification of RSV have changed over the last two decades. However, although clinical practice may have changed within the study period, polymerase chain reaction (PCR) tests have been an established diagnostic procedure for RSV for the entirety of the study period minimising the potential impact on the results.

The number of identified RSV cases in this study and the official reporting of RSV hospitalisation from Danish authorities do not match completely. The inclusion criteria were strict, to ensure that the identified RSV cases were in fact RSV cases. However, this results in some cases being excluded if they were not observed with one of the inclusion ICD-10 codes, or if they did not provide a PCR sample, confirming their RSV disease.

## Conclusion

The annual attributable cost of RSV episodes requiring hospitalisation in infants younger than six months of age is EUR 4.3 million in Denmark. Additionally, RSV generated EUR 2.1 million attributable cost in the year following the RSV hospitalisation. For many years, RSV has significantly burdened paediatric hospital departments, particularly during the winter months. With the availability of new RSV infant prevention options, should these preventive possibilities be recommended and reimbursed by health authorities, the overall burden of RSV is expected to be substantially reduced in the future.

## Data Availability

The data that support the findings of this study are available from Statistics Denmark’s Research Service. However, restrictions apply to the availability of these data, which were used under license/authorisation for the current study and so are not publicly available. Additional data analyses are available from the authors upon reasonable request and with permission of Statistics Denmark’s Research Service.

## Abbreviations

CI: Confidence interval
CIOMS: Council for International Organizations of Medical Sciences
CRS: The Civil Registration System
DKK: Danish currency (Danish Krone)
DRG: Diagnosis Related Groups
EUR: Euros
GP: General practitioner
ICD-10: International Statistical Classification of Diseases and Related Health Problems 10th Revision
MiBa: The Danish Microbiology Database
NPR: National Patient Register
PCR: Polymerase chain reaction
RSV: Respiratory syncytial virus
RUKS: Register of Selected Chronic Diseases
